# Radiomics features based on internal and marginal areas of the tumor for the preoperative prediction of microsatellite instability status in colorectal cancer

**DOI:** 10.3389/fonc.2022.1020349

**Published:** 2022-10-06

**Authors:** Yi Ma, Changsong Lin, Song Liu, Ying Wei, Changfeng Ji, Feng Shi, Fan Lin, Zhengyang Zhou

**Affiliations:** ^1^ Department of Radiology, Nanjing Drum Tower Hospital Clinical College of Nanjing Medical University, Nanjing, China; ^2^ Department of Bioinformatics, Nanjing Medical University, Nanjing, China; ^3^ Department of Research and Development, Shanghai United Imaging Intelligence Co., Ltd., Shanghai, China; ^4^ Department of Cell Biology, Nanjing Medical University, Nanjing, China

**Keywords:** microsatellite instability, radiomics, colorectal cancer, internal and marginal, computed tomography

## Abstract

**Objectives:**

To explore whether the preoperative CT radiomics can predict the status of microsatellite instability (MSI) in colorectal cancer (CRC) patients and identify the region with the most stable and high-efficiency radiomics features.

**Methods:**

This retrospective study involved 230 CRC patients with preoperative computed tomography scans and available MSI status between December 2019 and October 2021. Image segmentation and radiomic feature extraction were performed as follows. First, slices with the maximum tumor area (region of interest, ROI) were manually contoured. Subsequently, each ROI was shrunk inward by 1, 2, and 3 mm, respectively, where the remaining ROIs were considered as the internal region of the tumor (named as IROI1, IROI2, and IROI3), and the shrunk regions were considered as marginal regions of the tumor (named as MROI1, MROI2, and MROI3). Finally, radiomics features were extracted from each of the ROI. The intraclass correlation coefficient and least absolute shrinkage and selection operator method were used to choose the most reliable and relevant features of MSI status. Clinical, radiomics, and combined clinical radiomics models have been established. Calibration curve and decision curve analyses (DCA) were generated to explore the correction effect and assess the clinical applicability of the above models, respectively.

**Results:**

In the testing cohort, the radiomics model based on IROI3 yielded the highest average area under the curve (AUC) value of 0.908, compared with the remaining radiomics models. Additionally, hypertension and N stage were considered as clinically independent factors of MSI status. The combined clinical radiomics model achieved excellent diagnostic efficacy (AUC: 0.928; sensitivity: 0.840; specificity: 0.867) in the testing cohort, as well as favorable calibration and clinical utility by calibration curve and DCA analyses.

**Conclusions:**

The IROI3 model, which is based on a 3-mm shrink in the largest areas of the tumor, could noninvasively reflect the heterogeneity and genetic instability within the tumor. This suggests that it is an important biomarker for the preoperative prediction of MSI status. The model can extract more robust and effective radiomics features, which lays a foundation for the radiomics study of hollow organs, such as in CRC.

## Introduction

Colorectal cancer (CRC) ranks the second leading cause of cancer-related mortality worldwide, with approximately 700,000 deaths each year (1, 2). Microsatellite instability (MSI), reflects the spontaneous loss or gain of nucleotides from repetitive DNA tracts, which is present in about 15% of CRC cases (3, 4). This gene replication error is usually repaired by the DNA mismatch repair (MMR) system, which helps maintain genomic stability and reduces spontaneous mutations. MSI status could be subdivided into microsatellite instability-high (MSI-H), microsatellite instability-low (MSI-L) and microsatellite stable (MSS) according to the proportion of loci with MSI (5).

MSI status is a vital predictive factor for the screening, prognosis, and therapeutic decisions of CRC patients. First, identifying MSI status is helpful for screening patients for Lynch syndrome, the most common form of hereditary CRC (6). Second, MSI is an indicator of a good prognosis for stage II CRC patients. Compared with MSS CRC, the overall and disease-free survival of stage II CRC patients with MSI is significantly prolonged (7). Third, MSI is crucial for developing treatment strategies in patients with CRC. Some randomized controlled trials have shown that adjuvant chemotherapy can improve the overall survival rate of patients with MSS (8). But MSI patients can benefit from immunotherapy (9, 10) due to their multiple tumor mutation sites (11) and extensive immunogenicity (12).

However, methods to evaluate MSI status, including polymerase chain reaction (PCR) and immunohistochemistry (IHC), are invasive and costly (13, 14). Moreover, for inoperable patients, a small biopsy sample may not be sufficient to clarify the MSI status due to tumor heterogeneity (15, 16). Therefore, the development of a noninvasive, repeatable, and effective MSI prediction method before surgery is of great significance in treatment decision-making for patients with CRC.

Radiomics is an emerging technology for acquiring high-dimensional image data about tumor phenotypes and microenvironments that cannot be detected by the naked eye (17, 18). As a non-invasive and reproducible radiological biomarker, radiomics analysis has shown great potential in tumor staging (19), prognosis evaluation (20, 21), and KRAS status prediction (22) for CRC. Recent reports have shown that peritumoral and intratumoral radiomic features provide value for evaluating tumor biological behavior (23–26).

Considering the particularity of intestinal tumors, their edges are easily affected by air and feces in the intestinal cavity, resulting in artifacts (27, 28). We considered whether this would further affect the stability of the extracted radiomics features. Therefore, we retrospectively collected the clinical and MSI status information of patients with CRC. Through image segmentation and feature extraction from the internal and marginal regions of the tumors, we constructed a clinical model, seven radiomics models, and a visual nomogram to predict the preoperative MSI status. This study aimed to evaluate whether analyzing the imaging characteristics from different regions within the tumor is more helpful in predicting MSI status and identifying the region with the most stable and high-efficiency radiomics features.

## Materials and methods

### Patients

**Figure 1:**
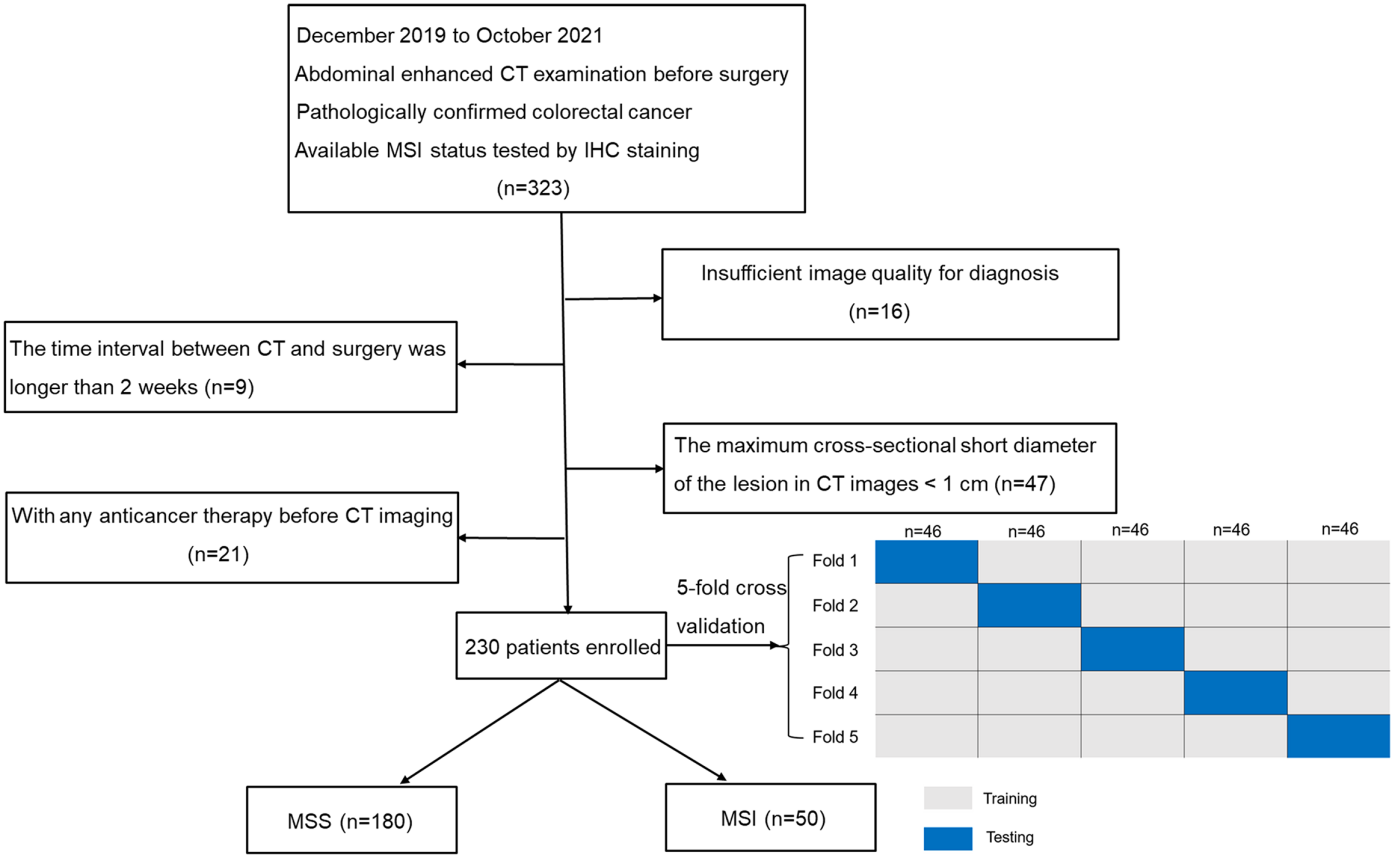
Flowchart of patient selection and grouping process. MSS Microsatellite stability, MSI Microsatellite instability.

This retrospective study was approved by the institutional ethical board at our hospital and exempted from informed consent. Data of 323 patients with CRC proved by surgical pathology were continuously collected from December 2019 to October 2021. Patient inclusion details were as follows: (a) patient underwent the abdominal enhanced computed tomography (CT) examination before surgery, (b) CRC confirmed by postoperative pathology, and (c) available MSI status tested by IHC. Patient exclusion details were as follows: (a) insufficient image quality to identify the tumor delineation with motion or metal artifacts (n = 16); (b) the interval between CT examination and operation exceeded 2 weeks (n = 9); (c) the maximum cross-sectional short diameter of the lesion was< 1 cm on CT images (n = 47); and (d) any anticancer therapy was performed before CT imaging (n = 21). The subject inclusion and exclusion criteria are presented in **Figure 1**. Finally, 50 patients with MSI and 180 with MSS CRC were enrolled in our study.

Clinical data, including sex, age, comorbidities, tumor location, TNM stage, histologic grade, carcinoembryonic antigen (CEA) level, carbohydrate antigen (CA) 125 level, and CA199 level, were abstracted from the medical records. These findings were unanimously confirmed by both clinicians.

### MSI status assessment

IHC staining of postoperative pathological tissues of CRC was first performed by a standard streptavidin-biotin-peroxidase procedure (29), then MSI status was determined by evaluating the results of the four MMR proteins (MLH1, MSH2, MSH6, and PMS2). Any of the four MMR protein negative expressions were identified as MSI, while all four MMR proteins positive expressions were identified as MSS (30).

### CT scan protocol

Patients underwent fasting for more than 4 h before the examination; during the scan, the patients were instructed to hold their breath, and each one received a flat scan first, followed by a three-phase enhanced scan. The scanning range was from the diaphragmatic apex to the bilateral suprapubic level. All studies were completed on the same CT scanner and received the same examination protocol. Parameters of the CT scan protocol are listed in **Table S1** of the **Supplementary Material**.

### Image segmentation and feature extraction

**Figure 2:**
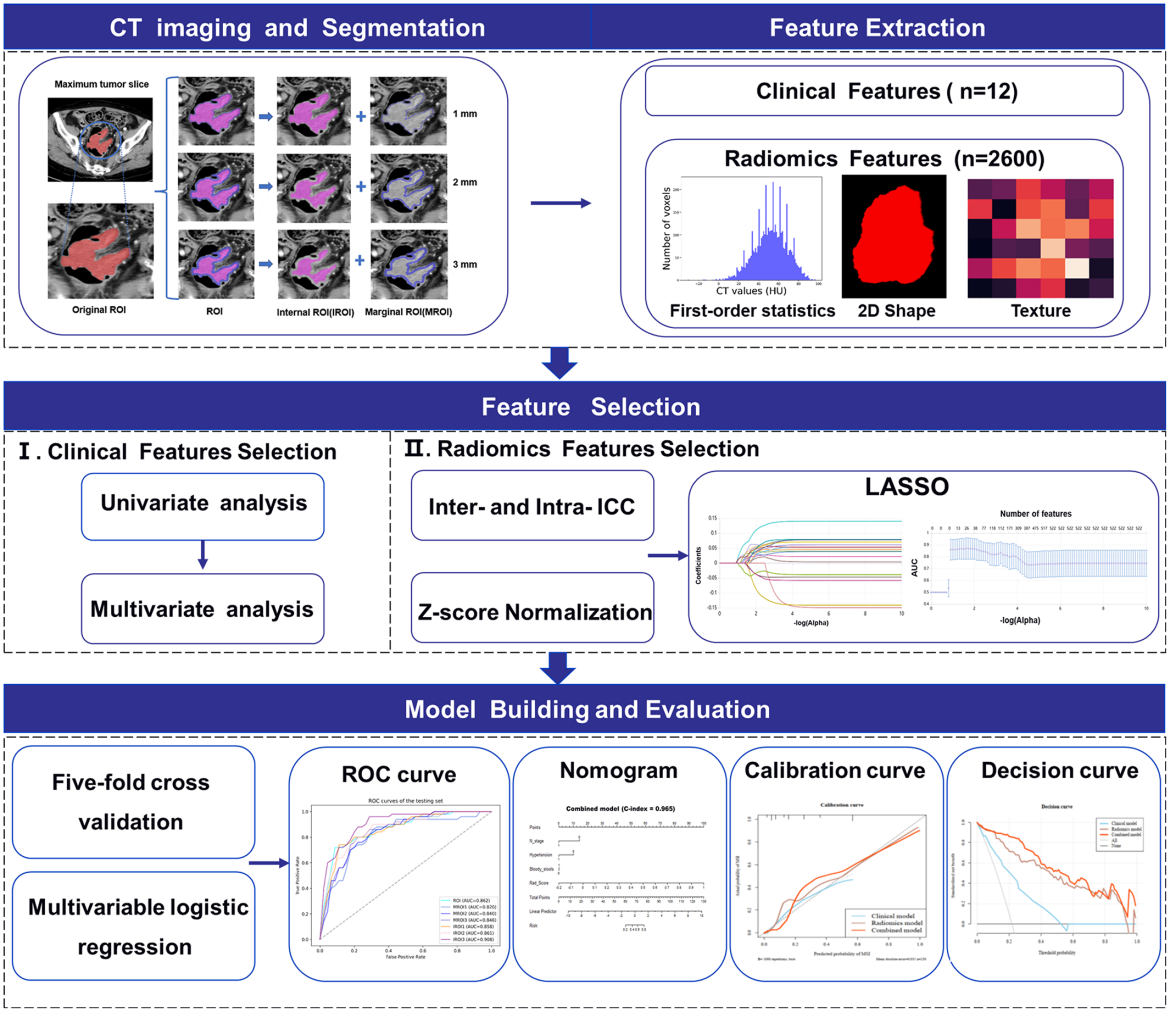
Workflow of MSI status prediction of colorectal cancer patients including image segmentation, feature extraction and selection, model building and evaluation.

**Figure 3:**
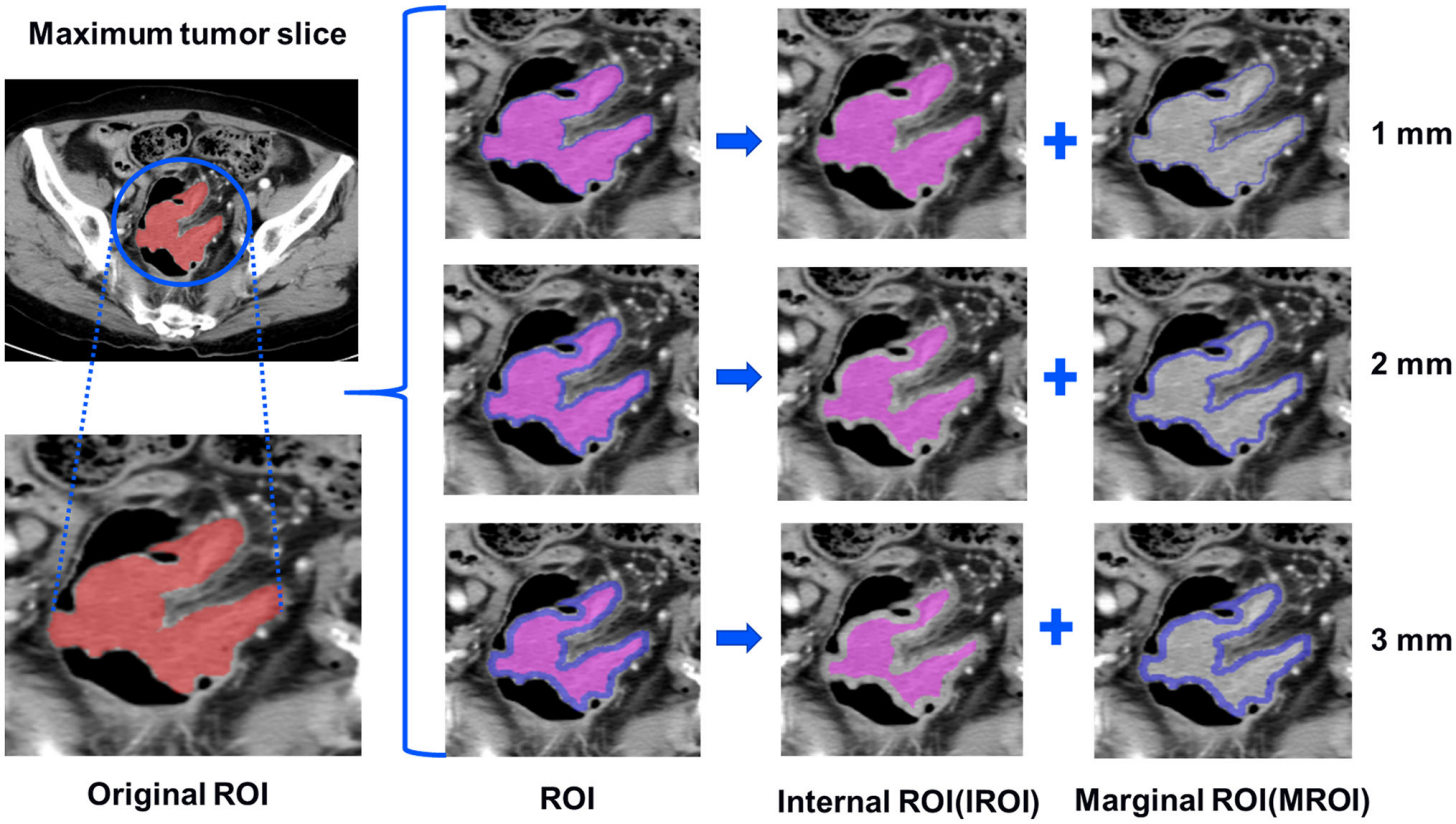
The tumors were segmented on venous phase, three groups of different marginal and internal regions were formed by retracting the tumor margin by 1, 2, and 3 mm on the representative section. The red areas represent the maximum slice of the tumor (ROI). The blue areas represent the rings with the thickness of 1-3mm in the marginal regions of the tumor (MROI), respectively. The hot-pink areas represent the remaining internal regions of the tumor (IROI) after removing the rings, respectively. ROI, regions of interest. MROI, the marginal areas of tumor. IROI, the interior areas of tumor.

The images of patients, which were selected from the picture archiving and communication system with a 5-mm venous layer thickness, were downloaded in DICOM format and uploaded to a research platform, the uAI Research Portal (Shanghai United Imaging Intelligence, Co., Ltd.). The flowchart contained image segmentation, feature extraction and selection, model building, and evaluation (**Figure 2**). All lesions were manually segmented by a senior abdominal radiologist (reader 1 [M.Y.] with 9 years of experience) blinded to MSI status. The slice with the maximum tumor area was selected, including bleeding and necrosis within the tumor, avoiding perienteric fat, vascular, air, and feces, and labeled as regions of interest (ROI). For the patients with multiple lesions, the largest lesion was selected for ROI delineation according to the endoscopic findings. We applied a set of morphological operations to the tumor region to analyze the information of the different areas inside the tumor. Since the outside of the tumor is mostly gas which may not provide useful information, we opted to use the morphological shrinkage operation (31) to automatically shrink the tumor boundary inward by 1, 2, and 3 mm. In this way, we define the remaining tumor regions as interior areas of the tumor (regarded as IROI1, IROI2, and IROI3), and assign the shrunken ring regions as the margin areas of the tumor (called MROI1, MROI2, and MROI3). Thus, the original tumor ROI is the sum of the marginal and corresponding internal regions **(** **Figure 3**).

Before Radiomics feature extraction, we first resampled all images into 1.5×1.5×1.5 mm^3^ to reduce any heterogeneity in acquisition voxel size. Second, we used an abdominal imaging window (window width (WW): 400, window level (WL): 40) for CT images normalization by min-max normalization method and adjusted the intensity range of each image to 0-255. Radiomics features were subsequently extracted from the widely used PyRadiomics (version 3.0.1) package (32), which contains seven feature categories: first-order statistics, shape, gray-level co-occurrence matrix (GLCM), gray-level dependence matrix (GLDM), gray-level size zone matrix (GLSZM), gray-level run length matrix (GLRLM), and neighboring gray-level tone difference matrix (NGTDM). Fourteen image filters were implemented with SimpleITK (version 2.1.0) package in Python were adopted (33), and Radiomics features were obtained from the filtered images as well as the original images. The image filters include additive Gaussian noise, binomial blur image, box mean, box sigma image, curvature flow, Laplacian sharpening, discrete Gaussian, mean, normalize, recursive Gaussian, shot noise, speckle noise, Laplacian of Gaussian and wavelet. Ultimately, 2,600 radiomics features were extracted from each annotation of the ROI, MROI1, MROI2, MROI3, IROI1, IROI2, and IROI3.

### Feature selection

Two months after image annotation, the above procedures were repeated by reader 1 and another abdominal radiologist (reader 2 [C.J.F.] with six years of experience) using 30 (34) randomly selected images. First, the ROIs of 30 patients were manually contoured, and the radiomic features of MROI1, MROI2, MROI3, IROI1, IROI2, and IROI3 were automatically obtained for each patient. The inter-/intra- delineator reproducibility was evaluated by the inter-/intra-class correlation coefficients (ICCs) with a pairwise correlation method, and features with both inter-class correlation coefficient and intra-class correlation coefficient greater than 0.75 are considered to have good or excellent reliability and used for subsequent feature selections. Z-score standardized normalization method was utilized to guarantee the comparability of different features by rescale the features of different levels into the same level. After the normalization, we used the least absolute shrinkage and selection operator (LASSO) algorithm (35), an approach that calculates the regression coefficients and successively shrinks them to avoid overfitting, to select the most helpful features in distinguishing between MSI and MSS. The corresponding coefficients were evaluated to calculate the Rad-score (RS) for each patient. RS was calculated using the following equation:


$$Rad-score\;=\sum_{i=1}^{n}C_{i}\;\times \;X_{i}+\;b$$

where *X_i_* is the ith feature, *C_i_* is the coefficient of the ith feature from the LASSO, *b* is the intercept of the LASSO regression algorithm, and *n* is the number of selected features.

### Model and nomogram construction

In this study, a stratified 5-fold cross-validation strategy was used to randomly but equally divided the patients into five partitions to ensure that the same percentage of each class (i.e., MSI/MSS) is preserved in each partition. One partition was taken each time without repetition as the testing cohort, and the remaining four partitions as the training cohort. The above steps were repeated five times to obtain five different sets of training-testing cohorts. The average value was obtained for a more stable and accurate sample evaluation **(** **Figure 1**).

The clinical parameters with *P*< 0.1 from the univariate analysis were enrolled into the multivariate regression analysis with *P*< 0.05 for identifying independent predictors of MSI status and constructing the clinical model (26, 36). Logistic regression analysis was used to develop seven radiomics models as well as a combined clinical radiomics model. The combined model was built with incorporating the RS of the optimal radiomics model (26) and the clinical independent predictors selected by clinical model, and it was presented in the form of an individualized nomogram.

### Statistical analysis

Clinical data were analyzed using the Mann–Whitney U test for continuous variables and χ^2^ test for categorical variables. A receiver operating characteristic (ROC) curve was used to evaluate the predictive effectiveness of each model. The areas under the ROC curve (AUC) of the radiomics models were quantified and compared. Calibration plots and decision curve analysis (DCA) were created to explore the correction effect and assess the clinical applicability of clinical, radiomics, and combined clinical radiomics models, respectively. The net reclassification index (NRI) was used to assess and compare the predictive power of the three models: NRI > 0 is a positive improvement, indicating that the predictive efficiency of the model has improved; NRI< 0 is a negative improvement, implying that the predictive efficiency of the model has decreased. All statistical analyses were conducted with the R software (version 3.5.2; http://www.Rproject.org) and IBM SPSS Statistics for Windows, version 26 (IBM Corp., Armonk, N.Y., USA). All statistical tests were two-sided, and statistical significance was set at *P*< 0.05.

## Results

### Patient characteristic

There were 230 CRC patients enrolled for analysis, consisting of 139 males (60.4%) and 91 females (39.6%) with an average age of 62.7 ± 11.8 years (range, 27–93 years); There were 203 patients with BMI records. The average BMI of the patients was 23.45 kg/m^2^ (range, 14.06 – 41.29 kg/m^2^). 180 patients (78.3%) were confirmed as having MSS and 50 (21.7%) were confirmed as having MSI. A comparison of the clinical characteristics between the two groups is summarized in **Table 1**. Univariate analysis revealed significant differences in patient gender, hypertension, tumor location, and N stage (all *P<* 0.05).

**Table 1:** Characteristics of patients [median (Q1, Q3) or no. (%)].

Variable	MSI (n = 50)	MSS (n = 180)	P_u_-value	P_m_-value
Gender			0.042	0.108
Male	24	115		
Female	26	65		
Age (years)	65 (52.75, 73)	63 (55, 71)	0.728	
Hypertension			0.019	0.014
Presence	12 (24.00%)	76 (42.22%)		
Absence	38 (76.00%)	104(57.78%)		
Diabetes			0.160	
Presence	10 (20.00%)	22 (12.22%)		
Absence	40 (80.00%)	158 (87.78%)		
Tumor location			< 0.000	0.222
Right colon	32 (64.00%)	58 (32.22%)		
Left colon	9 (18.00%)	34 (18.89%)		
Rectum	9 (18.00%)	88 (48.89%)		
Histologic grade			0.332	
Well	0 (0.00%)	4 (2.22%)		
Moderate	37 (74.00%)	144 (80.00%)		
Poor	13 (26.00%)	32 (17.78%)		
T stage			0.592	
T1~2	6 (12.00%)	27 (15.00%)		
T3~4	44 (88.00%)	153 (85.00%)		
N stage			0.001	0.001
N0	38 (76.00%)	87 (48.33%)		
N1~2	12 (24.00%)	93 (51.67%)		
M stage			1.000	
M0	50 (100.00%)	177 (98.33%)		
M1	0 (0.00%)	3 (1.67%)		
CEA (ng/ml)	2.34 (1.08, 5.44)	2.49 (1.27, 6.40)	0.514	
CA125 (U/ml)	8.90 (6.15, 16.95)	7.90 (5.00, 11.10)	0.069	0.457
CA199 (U/ml)	15.96 (7.40, 24.39)	12.54 (7.25, 32.52)	0.651	

Pu-value represents p-value derived from Mann-Whitney U test, and Pm-value is obtained from multivariable logistic regression analysis. P values less than.05 were considered as statistically significant.

CEA, carcinoembryonic antigen level; CA, carbohydrate antigen.

### Clinical model building

Logistic regression analysis identified hypertension [β= -0.971, OR = 0.379 (95% confidence intervals (CI), 0.175–0.822), *P* = 0.014] and N stage [β= -1.338, OR = 0.262 (95% CI, 0.123–0.561), *P* = 0.001] as independent factors of MSI status. A clinical model incorporating the above predictors had been developed and it produced moderate performance with an AUC of 0.695 (95% CI, 0.523–0.867) in the testing cohort. (**Supplementary Table S2**).

### Radiomics feature selection and model analysis

**Figure 4:**
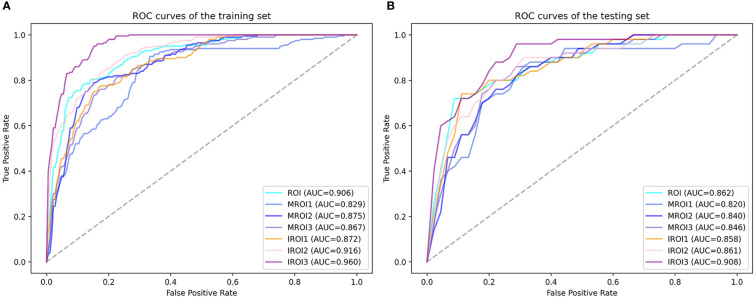
The receiver operating characteristic (ROC) curves of seven radiomics models in training cohort **(A)** and testing cohort **(B)**. IROI3 model [area under the curve (AUC) = 0.960 and 0.908 in the training and testing cohort, respectively] achieved better performance than the other radiomics models.

The features with low reproducibility (ICC values< 0.75) were removed as described earlier, so the number of features extracted from ROI MROI1, MROI2, MROI3, IROI1, IROI2, and IROI3 were reduced to 2069, 1260, 1485, 1600, 2029, 2003, and 1828, respectively. After Z-score normalization, the most representative radiomics features were screened by LASSO to build the logistic regression model. The number of selected features and the efficiency of the seven different radiomics models are listed in **Table 2**. This indicated that the established 7 radiomics models could perform well (all AUC ≥ 0.820) to predict the MSI status of CRC preoperatively. In the testing cohort, the internal regions of the tumor labeled as IROI1 [AUC: 0.858 (95% CI, 0.728–0.989)], IROI2 [AUC: 0.861 (95% CI, 0.751–0.969)], and IROI3 [AUC: 0.908 (95% CI, 0.821–0.991)] produced higher AUC values than the three corresponding marginal areas of tumor regarded as MROI1 [AUC: 0.820 (95% CI, 0.674–0.965)], MROI2 [AUC: 0.840 (95% CI, 0.714–0.961)], and MROI3 [AUC: 0.846 (95% CI, 0.722–0.969)]. In addition, by analyzing the internal regions of the tumor labeled as IROI1, IROI2, and IROI3, an improvement in predictive performance was observed. Moreover, the radiomics model based on IROI3 yielded the best predictive performance (accuracy: 0.813; sensitivity: 0.800; specificity: 0.817). The ROC analysis to assess the performance of different radiomics models is shown in **Figure 4**. Ultimately, IROI3 was chosen as the final radiomics model because of its best prediction ability compared to the remaining models. (**Figure 4**, **Table 2**). The features used to build the IROI3 model are shown in **Figure S1** in the **Supplementary Material**.

**Table 2:** Predictive performance of seven radiomics models in training and testing cohorts.

Model	Feature_num	Training cohort				Testing cohort			
		AUC (95% CI)	Accuracy	Sensitivity	Specificity	AUC (95% CI)	Accuracy	Sensitivity	Specificity
ROI	15	0.906 (0.857-0.957)	0.804	0.845	0.793	0.862 (0.729-0.990)	0.770	0.760	0.772
MROI1	19	0.829 (0.757-0.901)	0.740	0.775	0.731	0.820 (0.674-0.965)	0.730	0.760	0.722
MROI2	27	0.875 (0.819-0.932)	0.767	0.820	0.753	0.840 (0.714-0.961)	0.726	0.780	0.711
MROI3	11	0.867 (0.808-0.926)	0.788	0.790	0.788	0.846 (0.722-0.969)	0.778	0.760	0.783
IROI1	13	0.872 (0.816-0.930)	0.743	0.840	0.717	0.858 (0.728-0.989)	0.722	0.800	0.700
IROI2	26	0.916 (0.873-0.960)	0.811	0.855	0.799	0.861 (0.751-0.969)	0.770	0.800	0.761
IROI3	23	0.960 (0.938-0.986)	0.876	0.895	0.871	0.908 (0.821-0.991)	0.813	0.800	0.817

ROI, regions of interest. MROI, the marginal areas of tumor. IROI, the interior areas of tumor. AUC, area under the curve.

### Clinical application

**Figure 5:**
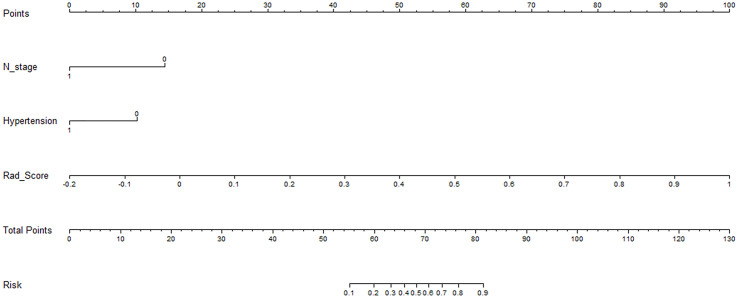
An individualized nomogram for preoperative predicting the status of MSI in colorectal cancer patients. The Nomogram was built based on the N stage, hypertension and the Rad-score of the optimal radiomics model.

**Figure 6:**
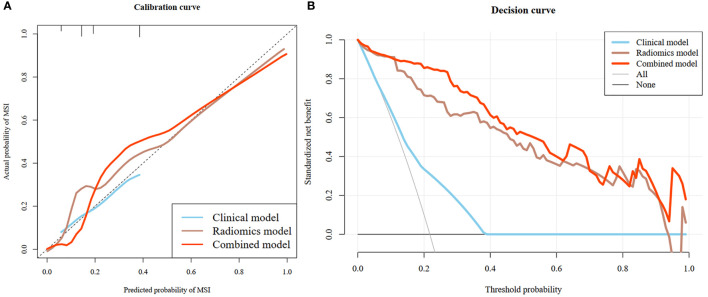
Calibration curves of the clinical model, radiomics model and combined model **(A)**. The diagonal dotted line indicated perfect prediction, and the solid lines indicated the prediction performance of the three models respectively. The closer to the dotted line, the better prediction of the model. The calibration curve showed the radiomics and combined models with favorable performance for predicting MSI status, which was better than clinical model. Decision curve analysis (DCA) of the three model **(B)**. The x-axis represented the threshold probability. The y-axis was the net benefit. The higher curve at any range of threshold probability was the best prediction to maximize the net benefit. The DCA indicated that the combined model provides a better clinical utility than the other two models.

The rad-score of IROI3 and selected clinically independent factors were used to develop a combined model based on logistic regression, which was presented as a quantitative nomogram (**Figure 5**). In the testing cohort, the combined model (AUC: 0.928 [95% CI, 0.860–0.991)] had a better performance than the clinical or radiomics models. NRIs further indicated significant improvements in the combined model compared to the clinical model (NRI: 0.490, *P<* 0.001), with no significant improvements in the combined model compared to the radiomics model (NRI: 0.090, *P* = 0.097) (**Table 3**). The calibration curve showed that the radiomics and combined models had a better agreement between observation and prediction to evaluate MSI status than the clinical model. The decision curves of the combined model gained the highest net benefit compared to the other two models at ranges of 12–70% for the radiomics model and 0–100% for the clinical model (**Figure 6**).

**Table 3:** Pairwise comparisons of AUCs of the clinical model, radiomics model, and combined model.

Cohorts	AUC (95% CI)			NRI_0vs.1_(P value)	NRI_0vs.2_(P value)	NRI_1vs.2_(P value)
	Clinical model (0)	Radiomics model (1)	Combined model (2)			
Training	0.691 (0.605-0.777)	0.960 (0.938-0.986)	0.977 (0.962-0.994)	0.458 (<0.001)	0.551 (<0.001)	0.093 (<0.001)
Testing	0.695 (0.523-0.867)	0.908 (0.821-0.991)	0.928 (0.860-0.991)	0.400 (<0.001)	0.490 (<0.001)	0.090 (0.097)

NRI, Net reclassification index.

## Discussion

In this retrospective study, radiomics features were extracted from different regions of the tumor for the prediction of MSI status to explore the effects of air and feces around the tumors on the model. We constructed three groups of different marginal and internal models by retracting the tumor margin by 1, 2, and 3 mm on the representative section. Seven radiomics models were developed to evaluate the MSI status. The clinical features were added to build a visual nomogram. Our results showed that the model based on the 3-mm adduction had the highest AUC value to predict the MSI state. The nomogram showed outstanding prediction with an AUC value of 0.928 in the testing cohort.

This study included 12 clinical indicators. The incidence of MSI was 21.74% (50/230), and it mostly occurred in the right colon at a rate of 64% (32/50), which is consistent with previous literature (37, 38). We also found that N stage and hypertension were clinically independent predictors and closely related to MSI status. Some studies (7, 39) have shown that patients with MSI CRC have a better prognosis, which may be associated with the lower rate of lymph node metastasis in the current study. The incidence rate of hypertension was lower in patients with MSI; these intriguing findings may help to clarify the status of MSI before operation and save costs. Although a recent study (40) based on 100 patients showed that there was no significant relationship between hypertension and MSI status, this conclusion needs to be further validated on a larger data.

Consistent with most relevant studies in recent years (41–44), we adopted the IHC method to determine MSI status. Although PCR is the gold standard for diagnosing MSI status, the operation process is complex and the cost is high. IHC has a high correlation with the detection results of PCR. It provides a cost-effective, sensitive (92.3%), and extremely specific (100%) method for screening for DNA mismatch repair defects (29).

CRC is surrounded by irrelevant information, such as air and feces in the intestinal cavity, and the radiomics features extracted from adjacent areas may be affected. Accordingly, in our study, the maximum cross-section of the lesion was delineated, and the adduction technique was used to analyze radiomics features from different intratumoral regions. The short diameters of the included tumors were > 1 cm, which not only optimize the clinical efficacy (45), but also avoid image annihilation during processing. The tumor margin was retracted by 1–3 mm respectively, and seven radiomics models were generated. By comparing the AUC values of the models, 23 quantitative radiomic features were selected to calculate the RS of the optimal radiomic model. Among them, 19 texture features were obtained from five categories of texture features (i.e., GLCM, GLDM, GLSZM, GLRLM, and NGTDM). These features were used as a measure of the grayscale non-uniformity of the images to reflect the inherent heterogeneity of the tumor. This finding was consistent with a previous study in which the commonest radiomics features to predict MSI status were the texture features (46).

Our research found that the model based on a 3 mm adduction was determined as the optimal radiomics model. The combined clinical radiomics model showed excellent prediction with an AUC of 0.928 in the testing cohort, which was higher than that reported in previous studies (41–44). Furthermore, the internal regions of the tumor produced a higher AUC value than the corresponding marginal areas of the tumor. The closer to the tumor center, the greater the improvement in the predictive performance.

The excellent ability of our model to predict MSI status may be explained as follows. Pathologically, MSI CRC tends to manifest as mixed morphological characteristics, such as mucinous, glandular, and solid components (47), which causes tumor heterogeneity and made it possible to enable the analysis of preoperative MSI status with radiomics. Moreover, the invasive ability of cancer cells differs between the center and edge of the tumor. A study by Zhao et al. (48) found that compared with cancer cells at the edge of the tumor, cancer cells in the center of the tumor were easier to metastasize and spread. Furthermore, the marginal areas of the tumor may be affected by adjacent air or feces. The generated artifacts may make the extracted features unstable, thus affecting the model performance. Compared to the radiomics model, we found that the performance of the combined model was not significantly improved after adding clinical features in the testing cohort (*P* = 0.097). This result also implied the independent value of the radiomics model we created in the preoperative prediction of MSI status in CRC patients.

In order to promote clinical practice, we built a radiomics nomogram based on the characteristics of radiology and clinical features to realize the non-invasive and individualized prediction of the preoperative MSI status of CRC patients by clinicians. Moreover, considering the influence of intestinal contents and feces around the colorectal cancer, we measured adduction of the primary lesions in 2D plane to determine the ROI and build models to predict the preoperative microsatellite status. In this study, the ROI region was an objective and quantitative analysis and processing based on the location of the primary lesion. The simplicity and repeatability of the prediction models were further verified in the testing cohort. Therefore, the results of this study provide a new method to determine ROI for future imaging radiomics research on CRC.

Nevertheless, this study still had several limitations. Firstly, it was a single-center study with limited samples. Hence, further verification is necessary by conducting an external and multicenter study. Second, there might have been a selection bias owing to the retrospective study. Third, all images were obtained using the same CT scanner, which might have affected the generalizability of our results. Fourth, for small tumors with a 3 mm adduction, the remaining internal area was too small, affecting the modeling performance, although we chose tumors with a short diameter greater than 1 cm to avoid image annihilation. Further exploration is needed to develop an equal-proportion adduction processing software according to the tumor size.

## Conclusion

Three different groups of marginal and internal models to predict MSI status, were constructed by adducting the tumor edge by 1–3 mm. Our study confirmed that a model based on the 3-mm adduction can noninvasively reflect tumor heterogeneity and genetic instability. It is an important biomarker for the preoperative prediction of MSI status in CRC patients. The model can extract more stable and effective radiomics features, which lays a foundation for the radiomics study of hollow organs, such as in CRC.

## Data availability statement

The raw data supporting the conclusions of this article will be made available by the authors, without undue reservation.

## Ethics statement

The studies involving human participants were reviewed and approved by Medical Ethics Committee of the Nanjing Drum Tower Hospital. Written informed consent for participation was not required for this study in accordance with the national legislation and the institutional requirements.

## Author contributions

Data curation, study design, manuscript writing, and manuscript approval were performed by YM and CL, they were accountable for all aspects of the work. CT data analysis and manuscript approval were performed by SL and CJ. Statistical analysis and manuscript approval were performed by YW and FS. FL and ZZ conceptualized and designed the study. All authors contributed to the article and approved the submitted version.

## Conflict of interest

YW and FS are employees of Shanghai United Imaging Intelligence Co., Ltd. The company has no role in performing the surveillances and interpreting the data.

The remaining authors declare that the research was conducted in the absence of any commercial or financial relationships that could be construed as a potential conflict of interest.

## Publisher’s note

All claims expressed in this article are solely those of the authors and do not necessarily represent those of their affiliated organizations, or those of the publisher, the editors and the reviewers. Any product that may be evaluated in this article, or claim that may be made by its manufacturer, is not guaranteed or endorsed by the publisher.
