# Supplementary material for: Radiomics features based on internal and marginal areas of the tumor for the preoperative prediction of microsatellite instability status in colorectal cancer
Source: Front Oncol. 2022 Oct 6;12:1020349. doi: 10.3389/fonc.2022.1020349 (PMC9583004; doi:10.3389/fonc.2022.1020349)
Supplement: Supplementary file 1 [file DataSheet_1.docx]

Supplementary Material

**Supplementary Tables**

**Table S1. The parameters of the CT scan protocol.**

| **CT protocol** | |
| --- | --- |
| Scanner | UIH uCT780, Shanghai, China |
| Tube voltage | 120 kVp |
| Tube current | 150 ~ 250 mA |
| Slice thickness | 5 mm |
| Slice interval | 5 mm |
| Reconstructed slice thickness | 1 mm |
| Reconstructed slice interval | 1 mm |
| Field of view | 350 × 350 mm |
| Image matrix | 512 × 512 |
| Rotation time | 0.5 seconds |
| Pitch | 0.9875 |
| Contrast agent | Omnipaque 350 mg I/mL, GE Healthcare |
| Flow rate | 3 mL/s |
| Dosage | 1.5 mL/kg |
| The arterial phase | 40 s |
| The venous phase | 70 s |
| The delayed phase | 180 s |

**Table S2. Predictive performance of the clinical model, radiomics model, and combined model in training and testing cohorts.**

| **Model** | **Training cohort** | | | | **Testing cohort** | | | |
| --- | --- | --- | --- | --- | --- | --- | --- | --- |
|  | **AUC (95% CI)** | **Accuracy** | **Sensitivity** | **Specificity** | **AUC (95% CI)** | **Accuracy** | **Sensitivity** | **Specificity** |
| **Clinical model** | 0.691  (0.605-0.777) | 0.628 | 0.700 | 0.608 | 0.695  (0.523-0.867) | 0.613 | 0.600 | 0.617 |
| **Radiomics model** | 0.960  (0.938-0.986) | 0.876 | 0.895 | 0.871 | 0.908  (0.821-0.991) | 0.813 | 0.800 | 0.817 |
| **Combined model** | 0.977  (0.962-0.994) | 0.907 | 0.970 | 0.889 | 0.928  (0.860-0.991) | 0.861 | 0.840 | 0.867 |

**Supplementary Figure**


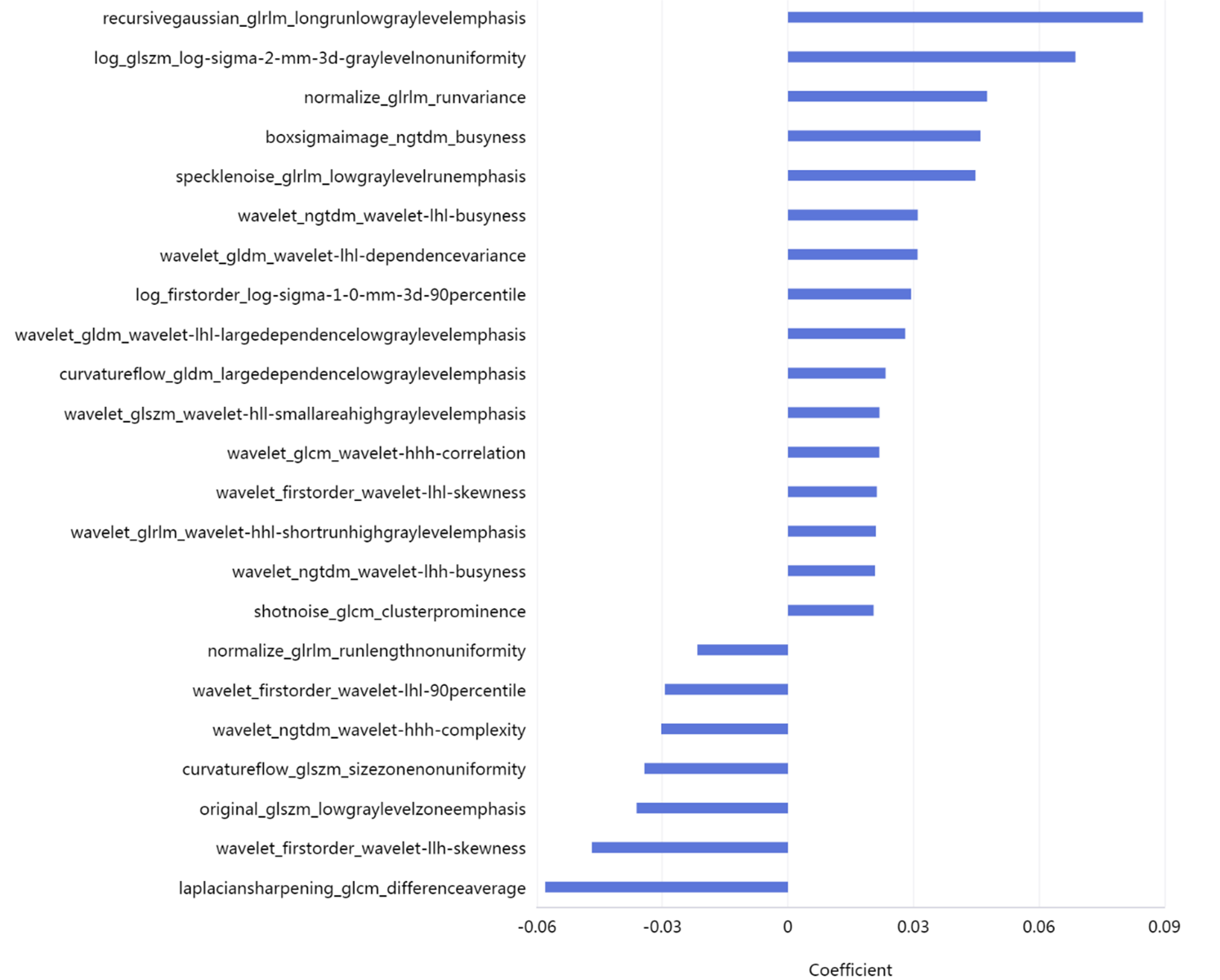


**Figure S1**. The 23 selected features of the final radiomics model. The 23 most predictive features were extracted from the IROI3 formed by 3mm adduction of tumor margin. The corresponding coefficients of the features were evaluated by the least absolute shrinkage and selection operator (LASSO) algorithm.
